# Characterization of the functional and transcriptomic effects of pro-inflammatory cytokines on human EndoC-βH5 beta cells

**DOI:** 10.3389/fendo.2023.1128523

**Published:** 2023-04-11

**Authors:** Caroline Frørup, Rebekka Gerwig, Cecilie Amalie Søndergaard Svane, Joana Mendes Lopes de Melo, Kristine Henriksen, Tina Fløyel, Flemming Pociot, Simranjeet Kaur, Joachim Størling

**Affiliations:** ^1^ Translational Type 1 Diabetes Research, Clinical Research, Steno Diabetes Center Copenhagen, Herlev, Denmark; ^2^ Faculty of Health and Medical Sciences, University of Copenhagen, Copenhagen, Denmark; ^3^ Department of Biomedical Sciences, University of Copenhagen, Copenhagen, Denmark

**Keywords:** pancreatic beta cells, type 1 diabetes, model system, inflammation, apoptosis, insulin, signaling, RNA-Seq

## Abstract

**Objective:**

EndoC-βH5 is a newly established human beta-cell model which may be superior to previous model systems. Exposure of beta cells to pro-inflammatory cytokines is widely used when studying immune-mediated beta-cell failure in type 1 diabetes. We therefore performed an in-depth characterization of the effects of cytokines on EndoC-βH5 cells.

**Methods:**

The sensitivity profile of EndoC-βH5 cells to the toxic effects of interleukin-1β (IL-1β), interferon γ (IFNγ) and tumor necrosis factor-α (TNFα) was examined in titration and time-course experiments. Cell death was evaluated by caspase-3/7 activity, cytotoxicity, viability, TUNEL assay and immunoblotting. Activation of signaling pathways and major histocompatibility complex (MHC)-I expression were examined by immunoblotting, immunofluorescence, and real-time quantitative PCR (qPCR). Insulin and chemokine secretion were measured by ELISA and Meso Scale Discovery multiplexing electrochemiluminescence, respectively. Mitochondrial function was evaluated by extracellular flux technology. Global gene expression was characterized by stranded RNA sequencing.

**Results:**

Cytokines increased caspase-3/7 activity and cytotoxicity in EndoC-βH5 cells in a time- and dose-dependent manner. The proapoptotic effect of cytokines was primarily driven by IFNγ signal transduction. Cytokine exposure induced MHC-I expression and chemokine production and secretion. Further, cytokines caused impaired mitochondrial function and diminished glucose-stimulated insulin secretion. Finally, we report significant changes to the EndoC-βH5 transcriptome including upregulation of the human leukocyte antigen (*HLA*) genes, endoplasmic reticulum stress markers, and non-coding RNAs, in response to cytokines. Among the differentially expressed genes were several type 1 diabetes risk genes.

**Conclusion:**

Our study provides detailed insight into the functional and transcriptomic effects of cytokines on EndoC-βH5 cells. This information should be useful for future studies using this novel beta-cell model.

## 1 Introduction

Cell models of human beta cells are important research tools for studying beta-cell biology and understanding beta-cell failure in diabetes. Most *in vitro* beta-cell research has been performed using immortalized rodent beta-cell lines such as rat INS-1 and mouse MIN6 (1). Although the use of these models has advanced our understanding of several aspects of beta-cell biology under normal and pathophysiological conditions relevant for diabetes, the phenotypes of rodent beta cells do not satisfactorily resemble native human beta cells. In 2011, Ravassard and colleagues successfully developed the first human beta-cell line, EndoC-βH1, derived from a human fetus (2). For more than a decade, this cell line has proved a useful human beta-cell model although still with functional limitations compared to native human beta cells, including lower insulin production and secretory capacity, a tumorigenic phenotype, and chronic infection with a xenotropic murine virus (3–7).

A new human beta-cell model, EndoC-βH5, has recently become commercially available (https://www.humancelldesign.com/). These cells demonstrate high insulin capacity and glucose responsiveness similar to native beta cells (8, 9). However, several important aspects of the EndoC-βH5 cells still await to be characterized including their functional responses and behavior under relevant diabetogenic conditions. For instance, it is highly relevant to establish the EndoC-βH5 cells’ sensitivity profile to pro-inflammatory cytokines which are mediators of beta-cell impairment in type 1 diabetes and found in pancreatic islet infiltrates of donors with type 1 diabetes and in animal models of type 1 diabetes (10, 11). The cytokines interleukin-1β (IL-1β), interferon-γ (IFNγ) and tumor necrosis factor-α (TNFα) are the main mediators of beta-cell death and are commonly used *in vitro* as a model of type 1 diabetes pathogenesis (12–14). Evidence also suggests that cytokines contribute to beta-cell impairment in type 2 diabetes (15). Hence, there is broad interest in obtaining a better understanding of how cytokines exert their detrimental effects on beta cells. In the present study, we therefore set out to thoroughly characterize the EndoC-βH5 cells’ response to cytokines at the functional, signaling, and transcriptomic levels.

## 2 Methods

### 2.1 Cell culture of EndoC-βH5

Frozen stocks of EndoC-βH5 cells were purchased from Human Cell Design (Toulouse, France). The EndoC-βH5 cells originate from fetal pancreatic rudiments and were generated from differentiation and immortalization by transduction with the SV40 Large T-antigen (SV40LT) and human telomerase reverse transcriptase (hTERT) transgenes (9, 16). The EndoC-βH5 cells have furthermore undergone de-immortalization, including removal of the SV40L and hTERT transgenes, as well as maturation steps to generate functional and mature human beta cells (8, 9). Upon thawing, cells had a viability between 74-91% (n=24). The EndoC-βH5 cells were seeded in appropriate plates coated with β-coat (Human Cell Design) and maintained in Ultiβ1 cell culture medium (Human Cell Design) containing 100 U/mL penicillin and 100 µg/mL streptomycin (Life Technologies). Based on the experimental setup, cells were seeded in 24-well (200,000 cells/well) or 96-well (50,000 cells/well) plates in duplicate and incubated in a humidified incubator at 37°C with 5% CO_2_. Media change was carried out every 2-3 days. Cells were incubated for up to 7 days in the presence or absence of recombinant human IL-1β (R&D systems, Minneapolis, MN, USA), recombinant human IFNγ (PeproTech, Rocky Hill, NJ, USA) and/or recombinant human TNFα (R&D Systems). For long-term treatments (4 and 7 days) with cytokines, the culture medium was replaced with fresh medium and cytokines every 2-3 days.

### 2.2 Cell death and viability

The ApoTox-Glo Triplex Assay (Promega, Madison, WI, USA) was used to measure live-cell protease activity, dead-cell protease activity, and caspase-3/7 activity as markers of viability, cytotoxicity, and apoptosis, respectively. The assay was used according to the manufacturer’s protocol. Luminescence and fluorescence were measured on an Infinite M200 PRO plate reader (Tecan, Männedorf, Switzerland).

### 2.3 Immunoblotting

Preparation of protein lysates, measurements of protein concentrations, and immunoblotting were performed as previously described (17). Antibodies used were anti-cleaved caspase-7 (#9491S, Cell Signaling, 1:500), anti-major histocompatibility complex (MHC)-I (the human leukocyte antigen (HLA)-A/B/C molecules (#ALX-805-711-C100, Enzo, Farmingdale, NY, USA, 1:2000), anti-phosho-c-Jun N-terminal kinase (P-JNK) (#9252, Cell Signaling, 1:1000), anti-nuclear factor of kappa light polypeptide gene enhancer in B-cells inhibitor α (IκBα) (#J1512, Santa Cruz Biotechnology, Dallas, TX, USA, 1:500), anti-phospho-signal transducer and activator of transcription 1 (P-STAT1) (7649S, Cell Signaling, 1:1000), anti-Tubulin (T8203, Sigma-Aldrich, St. Louis, MO, USA, 1:2000), anti-glyceraldehyde-3-phosphate dehydrogenase (GAPDH) (#9482, Abcam, Cambridge, UK, 1:5000) and secondary HRP-conjugated anti-mouse (#7076, Cell Signaling, 1:1000) or anti-rabbit (#7074, Cell Signaling, 1:2000) IgG antibodies. Tubulin or GAPDH was used as internal controls for normalization.

### 2.4 Immunofluorescence

Cells were seeded in black 96-well plates with clear bottom. After treatment for 48 hours, cells were fixed in 4% paraformaldehyde (Thermo Fisher) for 15 min at RT and permeabilized in 0.25% Triton X-100 (Sigma-Aldrich) for 20 min at RT. Blocking was done using 5% goat serum (Invitrogen) for 2 hours at RT. Staining of MHC-I (HLA-A/B/C molecules) was carried out using anti-MHC-I antibody (W6/32) (#ALX-805-711-C100, Enzo, 1:200 O/N at 4°C) and Alexa Fluor 594-conjugated anti-mouse IgG antibody (#A11005, Invitrogen, 1:2000 for 2 hours RT). Furthermore, apoptosis was assessed by the detection of fragmented DNA using the Click-iT-Plus TUNEL Assay (Invitrogen, Waltham, Massachusetts, USA) according to the manufactures protocol. Nuclei staining was done with 0.5 µg/mL DAPI (Sigma-Aldrich) or 5 µg/mL Hoechst 33342 (Invitrogen) in PBS. Fluorescence was measured using the ImageXpress PICO Automated Cell Imaging System (Molecular Devices (UK) Limited, Berkshire, UK) and analyzed with the acquisition software (CellReporterXpress 2.9.3). Fluorescence was visualized using a Nikon ECLIPSE Ti2 microscope (Nikon, Tokyo, Japan). Images were acquired at x20 and x60 magnification and prepared using FIJI software (version 1.49).

### 2.5 Real-time qPCR

Gene expression levels of mRNAs and long non-coding RNAs (lncRNAs) shown previously to be regulators of beta-cell death and cellular impairment (13, 18, 19) were investigated by real-time quantitative PCR (qPCR). RNA was extracted using the RNeasy Mini Kit (Qiagen, Hilden, Germany) and cDNA was synthesized using the iScript cDNA Synthesis Kit (Bio-Rad, Hercules, CA, USA). Real-time qPCR was carried out with TaqMan Assays and TaqMan Gene Expression Master Mix (Applied Biosystems, Waltham, MA, USA) using a CFX384 C1000 Thermal cycler (Bio-Rad). Relative expression levels were analyzed using the 2^-ΔΔCt^ method (20) with normalization to *GAPDH*. *GAPDH* was chosen as this was the most stable housekeeping gene measured in EndoC-βH5 cells by real-time qPCR among *GAPDH*, Peptidylprolyl isomerase A (*PPIA*), hypoxanthine Phosphoribosyltransferase 1 (*HPRT1*), beta-actin (*ACTB*) and *18S* ribosomal RNA (data not shown).

### 2.6 Chemokine measurements

The chemokines IFN-gamma-inducible protein 10 (IP-10)/C–X–C motif chemokine 10 (CXCL10), Thymus and activation-regulated chemokine (TARC)/CCL17, Eotaxin/CCL11, Eotaxin-3/CCL26, Macrophage Inflammatory Proteins (MIP)-1β/CCL3, MIP-1α/CCL4, IL-8/CXCL8, Monocyte chemoattractant protein (MCP)-1/CCL2, MCP-4/CCL13, and Macrophage-derived chemokine (MDC)/CCL22 were measured in the cell culture medium by the V-PLEX Chemokine Panel 1 Human Kit (Meso Scale Diagnostics LLC., Rockville, Maryland) using a MESO Quickplex SQ 120 instrument (Meso Scale Diagnostics).

### 2.7 Glucose-stimulated insulin secretion

Cells were exposed to cytokines for 48 hours prior to glucose-stimulated insulin secretion (GSIS), including 24 hours in standard Ultiβ1 cell culture medium and 24 hours in Ulti-ST starvation medium (Human Cell Design) with and without IL-1β, IFNγ and TNFα. Cells were washed twice and incubated for 60 min in βKrebs (Human Cell Design). Cells were then incubated for 40 min in βKrebs supplemented with 0 or 20 mM glucose. Supernatants were collected and analyzed for insulin. The cells were lysed in Cell Death Detection Lysis Buffer (Roche, Basel, Switzerland) for analysis of insulin content. Insulin was measured by ELISA (Mercodia, Uppsala, Sweden) and read on an Infinite M200 PRO plate reader (Tecan). Insulin was normalized to DNA content using CyQuant (Thermo Fisher Scientific) according to the manufacturer’s protocol.

### 2.8 Seahorse extracellular flux

Cells were seeded in pre-coated XFe96 Seahorse plates (40,000 cells/well). After cytokine exposure, cells were washed twice and transferred into 180 µl Krebs-Ringer-HEPES (KRH) buffer (135 mM NaCl, 3.6 mM KCl, 10 mM HEPES, 0.5 mM MgCl2, 1.5 mM CaCl2, 0.5 mM NaH2PO4, 2 mM glutamine, 0,1% (w/v) fatty acid free BSA (Sigma-Aldrich), pH adjusted to 7.4 at 37°C with NaOH). Cells were incubated for 1 hour at 37°C in a CO_2_-free incubator prior to the experiment. After basal measurements, 11 mM glucose was injected to record respiratory response. Thereafter, 2 µM oligomycin (Sigma-Aldrich) was injected to inhibit ATP synthase and determine respiratory efficiency. Finally, 2 µM rotenone (Sigma-Aldrich), 2 µM antimycin A (Sigma-Aldrich), and 2.5 µM Hoechst 33342 was injected to inhibit mitochondrial respiration and stain for cell nuclei. Cell plates were immediately scanned using an ImageXpress PICO for cell count and quantified using the CellReporterXpress software. All data was normalized to cell count and corrected for non-mitochondrial respiration.

### 2.9 RNA sequencing

RNA-Seq libraries were sequenced on a DNBseq platform to produce 100 bp long paired-end reads. An average of ~40 million reads per replicate (EndoC-βH5 n=4) were obtained after pre-processing and QC (removal of low-quality reads and adapter sequences) using SOAPnuke software (21). Reads were aligned using TopHat (version 2.1.1) (22) to GChr38 genome with default parameters. Afterwards, reads were assigned to Ensembl version 107 gene annotation using htseq-count (version 0.9.1) (23) with default parameters. Minimum gene expression was defined based on a CPM cut-off of 0.5 in at least 4 samples. Differential gene expression analysis was performed on the two groups using the QL F-test in edgeR. Differentially expressed mRNAs and lncRNAs were identified using a cut-off of abs(log2FC) >0.5 and an FDR-adjusted p-value <0.05. We extracted the gene expression of pinpointed candidate genes from a total of 152 loci recently shown to be associated with type 1 diabetes (24). For cytokine receptor expression evaluation, TPM (transcripts per million) values were calculated for within-sample comparison of gene expression levels. All statistical analyses were conducted using Bioconductor packages in R.

### 2.10 Pathway analysis

Core analysis was performed using the Ingenuity Pathway Analysis (IPA) software (Qiagen, Valencia, CA, USA) on the differentially expressed genes to predict mechanistic and functional gene relationships, and identify top canonical pathways and molecular and cellular functions (24). The summarized upstream regulators, diseases, and mechanisms for the differentially expressed genes were visualized as a network and grouped using the IPA Path Designer Module.

### 2.11 Statistical analysis

Data are presented as means ± SEM. Graphs were constructed using GraphPad Prism 9. Statistical analyses were performed using one- or two-way ANOVA and/or one- or two-tailed paired t-test where appropriate. P-values <0.05 were considered statistically significant. Multiple testing was corrected using the Benjamini-Hochberg's correction.

## 3 Results

### 3.1 Cytokine sensitivity profile of EndoC-βH5 cells

**Figure 1:**
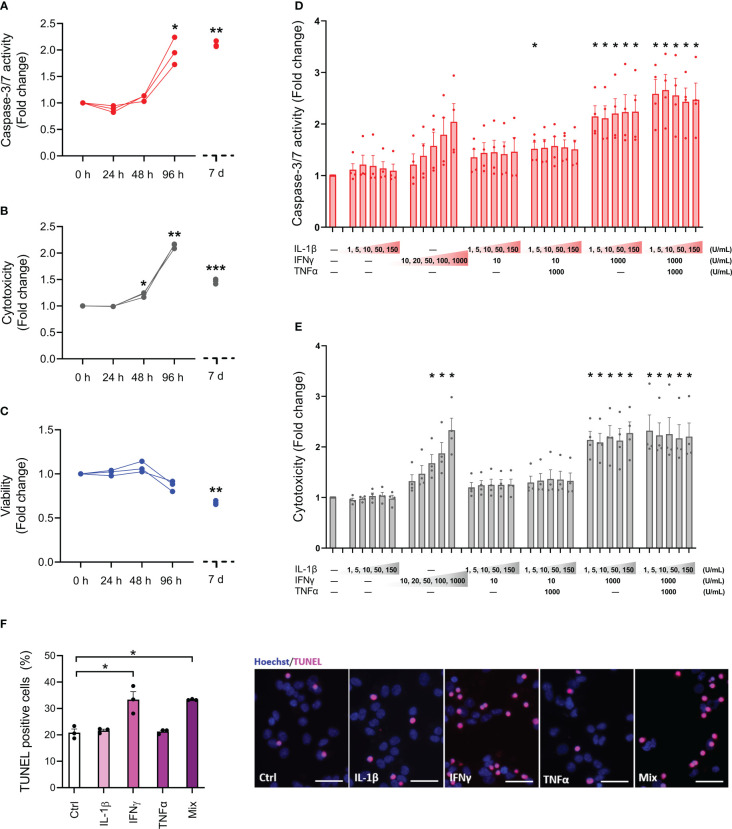
Cytokine sensitivity profile of EndoC-βH5 cells. **(A)** Caspase 3/7 activity, **(B)** cytotoxicity and **(C)** viability after exposure to cytokines (50 U/mL of IL-1β, 1000 U/mL IFNγ and 1000 U/mL TNFα) for 0 h, 24 h, 48 h, 96 h and 7 days. Data are means ± SEM (n=3), *p<0.05; **p<0.01. **(D)** Caspase-3/7 activity and **(E)** cytotoxicity after exposure to IL-1β, IFNγ and TNFα at the indicated concentrations for 96 h. Data are means ± SEM (n=4). Adjusted p-values *p<0.05. **(F)** TUNEL assay of EndoC-βH5 cells after exposure to cytokines (50 U/mL IL-1β, 1000 U/mL IFNγ and 1000 U/mL TNFα) alone or in combination (Mix) for 96 h. Representative immunofluorescent images of TUNEL (pink) and nuclei staining with Hoechst 33342 (blue). Scalebar 50 μm (x20). Data are presented as % TUNEL positive to total nuclei count, with means ± SEM (n=3), *p<0.05. Ctrl, untreated control.

To determine the sensitivity profile of the EndoC-βH5 cells, we initially examined the effects of cytokine exposure for 24, 48 and 96 hours, and after 7 days, using cytokine concentrations previously used by us and others on isolated human islets and EndoC-βH1 cells (50 U/mL of IL-1β, 1000 U/mL IFNγ and 1000 U/mL TNFα) (25, 26). Between 48 and 96 hours of cytokine exposure, caspase-3/7 activity and cytotoxicity were elevated and reached a ~2-fold increase after 4 days of cytokine exposure (**Figures 1A, B**). After 7 days of cytokine exposure, the caspase-3/7 activity and cytotoxicity were not further increased compared to 96 hours of cytokine treatment (**Figures 1A, B**). However, the viability of the cells declined with prolonged exposure to cytokines (**Figure 1C**). Microscopic inspection of cell morphology showed severe changes and detachment of cells after 7 days of cytokine exposure indicative of extensive cell death (**Supplementary Figure S1**).

Next, we studied the responses to various doses of individual and combinations of cytokines after 96 hours of exposure. As seen in **Figures 1D, E**, caspase-3/7 activity and cytotoxicity were dose-dependently induced by IFNγ alone. In contrast, IL-1β alone had no effects. When combined with IFNγ, neither IL-1β nor TNFα had synergistic effects on IFNγ-induced cell death. The reduction in cell viability by cytokine exposure was modest at this time point and did not reach statistical significance in any of the conditions (**Supplementary Figure S2**). These results indicate that only IFNγ exerts death-promoting and toxic effects on EndoC-βH5 cells.

End-stage apoptosis was evaluated by DNA fragmentation labelling with TUNEL. After 96 hours of cytokine exposure, end-stage apoptosis was elevated by ~1.6-fold in cells treated with either IFNγ alone or a combination of IL-1β, IFNγ, and TNFα (**Figure 1F**). We observed around 20% TUNEL-positive cells in the untreated control condition indicative of a relatively high basal apoptosis rate (**Figure 1F**). These results further support that IFNγ is the main cytotoxic cytokine among the tested ones in EndoC-βH5 cells.

### 3.2 Cytokine signal transduction in EndoC-βH5 cells

**Figure 2:**
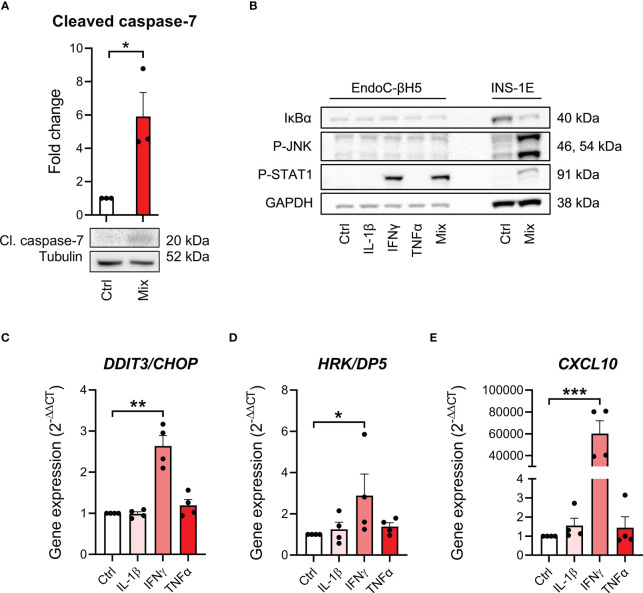
Cytokine-induced signal transduction in EndoC-βH5 cells. **(A)** Immunoblotting of cleaved caspase 7 following exposure to cytokines (50 U/mL IL-1β, 1000 U/mL IFNγ and 1000 U/mL TNFα) (Mix) for 96 h. Tubulin was used as loading control (n=3). **(B)** Immunoblotting of IκBα, phosphorylated STAT1, and phosphorylated JNK following exposure to cytokines (50 U/mL IL-1β, 1000 U/mL IFNγ and 1000 U/mL TNFα) alone or in combination (Mix) for 30 min. GAPDH was used as loading control. Representative image (n=3). Lysates from INS-1E cells with/without cytokine exposure were used as positive control. Gene expression of **(C)** *DDIT3*/*CHOP*, **(D)** *HRK*/*DP5* and **(E)** *CXCL10* in cells exposed to individual cytokines for 48 h. *GAPDH* was used as housekeeping gene. Data are means ± SEM (n=4), *p<0.05, **p<0.01, ***p<0.001. Ctrl, untreated control.

To study cytokine signal transduction in EndoC-βH5 cells, we investigated key signaling pathways of each cytokine. We first confirmed that cytokine exposure leads to apoptotic caspase activation shown by immunoblotting of cleaved, activated caspase 7 (**Figure 2A**). We then looked at proximal cytokine signal transduction by immunoblotting of the cellular content of activated, phosphorylated signal transducer of activated transcription 1 (P-STAT1) as a marker of IFNγ signaling, and P-JNK, and inhibitory κBα (IκBα) as markers of IL-1β and TNFα signaling (27). Cytokine treatment for 30 min strongly enhanced P-STAT1, whereas no effects were seen on P-JNK and IκBα (**Figure 2B**). INS-1E cell lysates were used as positive control and showed degradation of IκBα, elevated P-STAT1 and P-JNK after 30 min of cytokine exposure (**Figure 2B**).

A distal signaling event in cytokine-exposed beta cells is the induction of endoplasmic reticulum (ER) stress (13). Individual cytokine exposure showed that only IFNγ induced ER stress as revealed by increased expression of the ER stress markers DNA damage-inducible transcript 3 (*DDIT3*)/C/EBP homologous protein (*CHOP*) (28) and Harakiri (*HRK*)/Death Protein 5 (*DP5*) after 48 hours of cytokine exposure (29) (**Figures 2C, D**). Furthermore, increased expression of *CXCL10*, known as a key event in IFNγ signaling in beta-cell dysfunction and apoptosis (30), was greatly induced by IFNγ exposure (**Figure 2E**). These results suggest that at the time point examined, only IFNγ is capable of inducing intracellular signaling in EndoC-βH5 cells.

### 3.3 MHC-I expression in EndoC-βH5 cells

**Figure 3:**
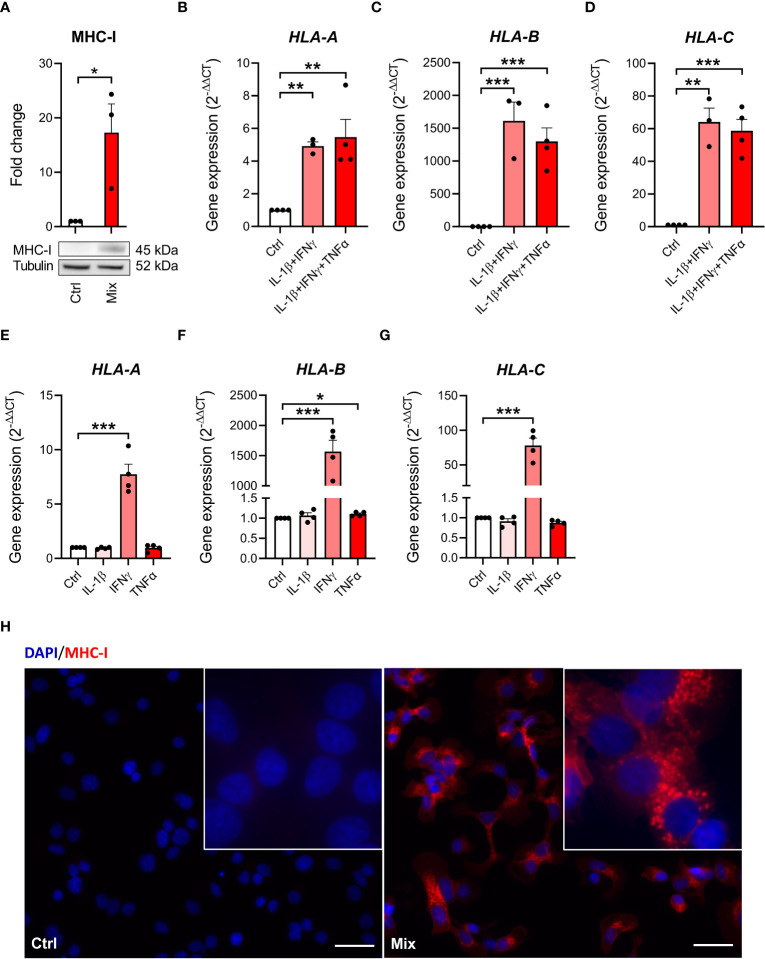
Cytokine-induced upregulation of MHC-I in EndoC-βH5 cells. **(A)** Immunoblotting of MHC-I in cells exposed to cytokines (50 U/mL IL-1β, 1000 U/mL IFNγ and 1000 U/mL TNFα) (Mix) for 96 h. Tubulin was used as a loading control. Data are means ± SEM (n=3). Gene expression of **(B)** *HLA-A* **(C)** *HLA-B* and **(D)** *HLA-C* in cells exposed to two or three cytokines for 48 h, and gene expression of **(E)** *HLA-A* **(F)** *HLA-B* and **(G)** *HLA-C* in cells exposed to individual cytokines for 48 h. *GAPDH* was used as housekeeping gene. Data are means ± SEM (n=3-4). **(H)** Immunofluorescence staining of MHC-I (red) and DAPI nuclei staining (blue) in EndoC-βH5 cells after 48 h of cytokine exposure. Scale bars indicate 50 μm (20x); zoom (60x). Representative images are shown. *p<0.05, **p<0.01, ***p<0.001. Ctrl, untreated control.

We next investigated MHC class I presence on EndoC-βH5 cells, a hallmark of immune-mediated beta-cell destruction in type 1 diabetes (18, 27, 31), in the EndoC-βH5 cells in response to cytokines. By immunoblotting, we found that MHC-I protein was induced after 96 hours of cytokine treatment (**Figure 3A**). Consistent with this, cytokines upregulated the expression of *HLA-A/B/C* which encode MHC-I (**Figures 3B–D**). Again, these effects were driven exclusively by IFNγ (**Figures 3E–G**). Induction of MHC-I was confirmed by immunofluorescence staining (**Figure 3H**). These findings demonstrate that IFNγ upregulate MHC-I in EndoC-βH5 cells.

### 3.4 Cytokine-induced secretion of chemokines from EndoC-βH5 cells

**Figure 4:**
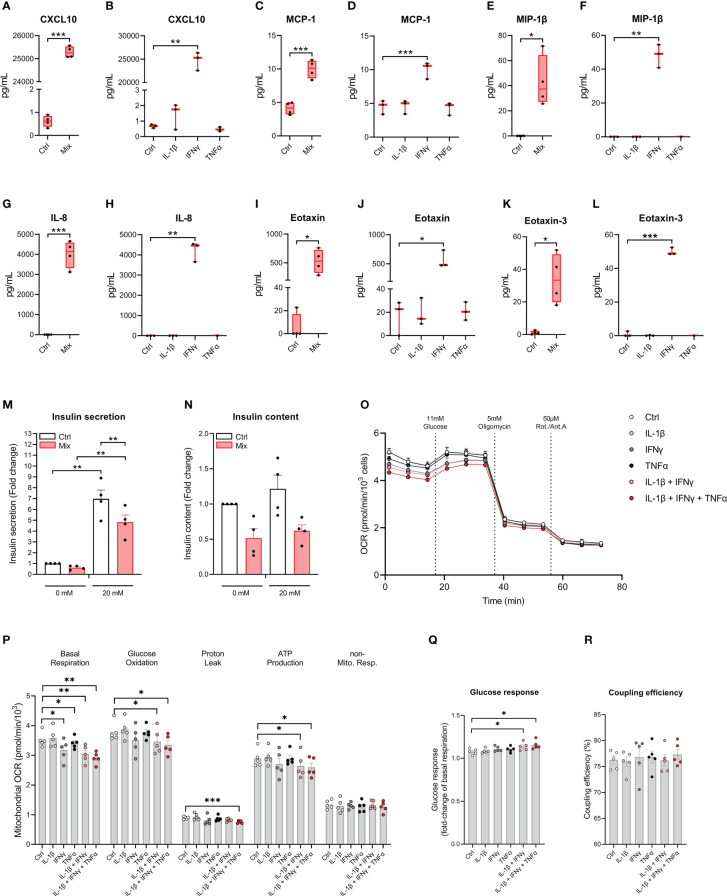
Cytokine-induced secretory and functional changes in the EndoC-βH5 cells. Accumulated chemokines in the culture medium from EndoC-βH5 cells after 48 h of cytokine treatment (50 U/mL IL-1β, 1000 U/mL IFNγ and 1000 U/mL TNFα) in combination (Mix) or as individual cytokines, respectively: **(A, B)** IP-10/CXCL10, **(C, D)** MCP-1/CCL2, **(E, F)** MIP-1β/CCL3, **(G, H)** IL-8/CXCL8, **(I, J)** Eotaxin/CCL11 and **(K, L)** Eotaxin-3/CCL26. Data are mean pg/mL with median and 5/95 percentiles (n=3-4). **(M)** Glucose-stimulated insulin secretion and **(N)** insulin content after 48 h of cytokine treatment (50 U/mL IL-1β, 1000 U/mL IFNγ and 1000 U/mL TNFα) (Mix) in response to 0 mM or 20 mM glucose stimulation. Data was normalized to DNA content. Data are means ± SEM (n=4). Mitochondrial respiration in untreated cells and cells exposed to cytokines (50 U/mL IL-1β, 1000 U/mL IFNγ and 1000 U/mL TNFα) alone or in combinations, as indicated, for 48 h. **(O)** oxygen consumption rate (OCR) traces normalized to cell number per well. **(P)** Analysis of mitochondrial parameters from OCR traces, corrected for non-mitochondrial respiration. **(Q)** Glucose response of respiration as fold-change of basal mitochondrial respiration. **(R)** Coupling efficiency calculated as the oligomycin-sensitive fraction of glucose-stimulated mitochondrial respiration. Results are means ± SEM (n=5), *p<0.05, **p<0.01, ***p<0.001. Ctrl, untreated control.

We examined if cytokines increase the secretion of chemokines which play important roles in the immune cell – beta-cell crosstalk in type 1 diabetes (32). The accumulated levels of 10 chemokines were measured in the cell culture medium after 48 hours of exposure to IL-1β, IFNγ, and TNFα in combination or alone. All chemokines measured were increased in response to the treatment with the cytokine mixture, and for treatment with the individual cytokines, IFNγ was the sole cytokine capable of increasing chemokine secretion (**Figures 4A–L** and **Supplementary Figures S3A–H**). These data show that EndoC-βH5 cells secrete chemokines upon treatment with IFNγ.

### 3.5 Cytokines impair glucose-stimulated insulin secretion in EndoC-βH5 cells

To investigate the impact of cytokines on the insulin secretory capacity of EndoC-βH5 cells, GSIS experiments were performed following exposure to IL-1β, IFNγ and TNFα for 48 hours. In untreated control cells, there was a significant increase in GSIS corresponding to a fold change of >6 (**Figure 4M**). This induction was diminished by ~30% by cytokine exposure (**Figure 4M**). The effect correlated with tendencies towards reduced cellular insulin content (**Figure 4N**) and decreased expression of *INS* and *PDX1* (**Supplementary Figures S4A, B**). These results demonstrate that cytokines cause functional impairment of EndoC-βH5 cells.

### 3.6 Cytokines alter mitochondrial function in EndoC-βH5 cells

To investigate the effects of cytokines on mitochondrial function of EndoC-βH5 cells, we measured the oxygen consumption rate (OCR) using Seahorse extracellular flux technology following cytokine treatments (**Figure 4O**). Basal respiration decreased after cytokine exposure in all conditions except IL-1β alone (**Figure 4P**). Glucose oxidation, proton leak and ATP production were decreased in response to treatment with a combination of the cytokines. The glucose response was enhanced in cells exposed to the combined cytokines, as compared to control (**Figure 4Q**). No effects were observed for coupling efficiency (**Figure 4R**). The results suggest that cytokines, and particularly IFNγ, alter mitochondrial respiration in EndoC-βH5 cells.

### 3.7 Cytokines induce transcriptomic changes in EndoC-βH5 cells

**Figure 5:**
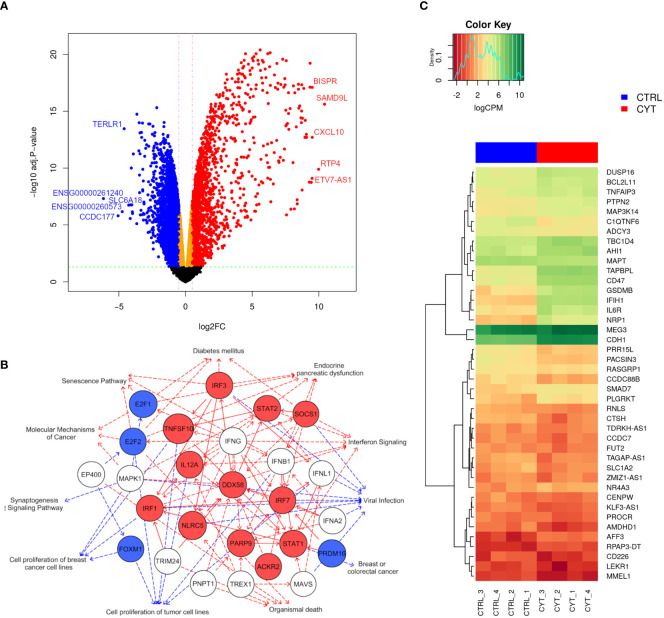
Cytokines modulate a large part of the gene expression profile of EndoC-βH5 cells. RNA sequencing of 4 replicates of untreated control cells (CTRL_1-4) or cells exposed to cytokines (50 U/mL IL-1β, 1000 U/mL IFNγ and 1000 U/mL TNFα) for 48 h. **(A)** Volcano plot showing differentially expressed genes after exposure to cytokines compared to untreated cells. The horizontal line represents the adjusted p-value and the vertical line represent fold change cut-offs. The red and blue dots represent the upregulated and downregulated genes, respectively. Top 5 most up- and downregulated mRNAs/lncRNAs ranked based on log-fold changes (logFC) are shown in the plot. **(B)** Graphical summary network representing the most significant upstream regulators, diseases, functions, and pathways, and their relations, as predicted by the IPA Core analysis. Red edges: predicted activated pathways, blue edges: predicted inhibited pathways. Solid lines represent direct relationships and dashed lines represent indirect relationships. Red nodes: upregulated, blue nodes: downregulated, white nodes: non-affected, based on differential expression from dataset. **(C)** Heat map and hierarchical clustering of the 42 differentially expressed candidate genes mapping to type 1 diabetes loci.

We next studied the impact of cytokines on the global gene expression profile of EndoC-βH5 cells. Cells were treated with or without IL-1β, IFNγ and TNFα for 48 hours followed by stranded RNA sequencing. On average, library sizes of ~40 million reads per sample were obtained (**Supplementary Figure S5A**). Multidimensional scaling (MDS) analysis showed a high separation between control and cytokine-treated samples (**Supplementary Figure S5B**). A total of 16,525 genes were detected of which more than one-third (6,000 genes) were differentially expressed in response to cytokines (**Figure 5A**; cut off of abs(log2FC) >0.5 and FDR adjusted p-value <0.05). The top 10 up- and downregulated coding (mRNA) and lncRNA genes are shown in **Table 1**. As lncRNAs are emerging as important regulators of cellular functions and disease, we wished to validate the expression of selected lncRNAs with roles in diabetes, e.g. taurine-upregulated gene 1 (*TUG1*), *LNC78*/TCL1 upstream neural differentiation-associated RNA (*TUNAR*), maternally expressed gene 3 (*MEG3*), growth arrest-specific transcript 5 (*GAS5*), and *LINC25*/*LINC01370*, known for their regulation of transcription factors and/or effects on beta-cell function and apoptosis (33–37). EndoC-βH5 cells exposed to cytokines for 48 hours showed significantly decreased expression of the lncRNAs *TUG1* and *TUNAR* (**Supplementary Figures S6A, B**). The lncRNAs *MEG3* and *GAS5* were not significantly affected by cytokine exposure (**Supplementary Figures S6C, D**). *LINC*-25 was not expressed (data not shown).

**Table 1:** Ten most up- and downregulated mRNAs and lncRNAs ranked based on log-fold changes (logFC) from RNA sequencing of untreated EndoC-βH5 cells or cells exposed to cytokines (50 U/mL IL-1β, 1000 U/mL IFNγ and 1000 U/mL TNFα) for 48 h.

mRNAs	Gene ID	Gene name	Chr.	logFC	p-value	FDR
**10 most upregulated**	ENSG00000177409	*SAMD9L*	7	10.43	1.41^-18^	2.42^-16^
	ENSG00000136514	*RTP4*	3	9.98	4.46^-12^	1.27^-10^
	ENSG00000169245	*CXCL10*	4	9.50	2.8^-15^	1.96^-13^
	ENSG00000156219	*ART3*	4	9.43	9.74^-11^	1.81^-09^
	ENSG00000169248	*CXCL11*	4	9.35	5.83^-23^	6.03^-20^
	ENSG00000090339	*ICAM1*	19	9.34	2.68^-20^	7.64^-18^
	ENSG00000130303	*BST2*	19	9.08	2.82^-15^	1.97^-13^
	ENSG00000162645	*GBP2*	1	8.99	6.28^-17^	6.33^-15^
	ENSG00000117228	*GBP1*	1	8.96	2.84^-15^	1.97^-13^
	ENSG00000173110	*HSPA6*	1	8.87	3.78^-20^	9.76^-18^
**10 most downregulated**	ENSG00000267909	*CCDC177*	14	-4.75	9.57^-08^	6.77^-07^
	ENSG00000164363	*SLC6A18*	5	-4.26	2.06^-08^	1.79^-07^
	ENSG00000100079	*LGALS2*	22	-4.15	4.77^-07^	2.72^-06^
	ENSG00000086967	*MYBPC2*	19	-4.12	1.95^-08^	1.71^-07^
	ENSG00000126861	*OMG*	17	-4.02	1.97^-08^	1.72^-07^
	ENSG00000205022	*PABPN1L*	16	-3.99	1^-07^	7.04^-07^
	ENSG00000187783	*TMEM72*	10	-3.98	1.46^-07^	9.83^-07^
	ENSG00000154134	*ROBO3*	11	-3.91	1.27^-13^	5.7^-12^
	ENSG00000135312	*HTR1B*	6	-3.66	1.58^-06^	7.71^-06^
	ENSG00000183379	*SYNDIG1L*	14	-3.65	1.49^-17^	1.82^-15^
lncRNAs	Gene ID	Gene name	Chr.	logFC	p-value	FDR
**10 most upregulated**	ENSG00000282851	*BISPR*	19	9.50	2.84^-20^	7.97^-18^
	ENSG00000224666	*ETV7-AS1*	6	9.49	3.69^-11^	7.9^-10^
	ENSG00000289582	*–*	1	9.28	9.19^-11^	1.72^-09^
	ENSG00000206337	*HCP5*	6	7.74	4.79^-16^	3.9^-14^
	ENSG00000256262	*USP30-AS1*	12	7.52	5.92^-18^	8.09^-16^
	ENSG00000272941	*–*	7	6.93	9.69^-21^	3.37^-18^
	ENSG00000273272	*–*	22	6.40	9.34^-09^	9.14^-08^
	ENSG00000261618	*LINC02605*	8	6.16	1.34^-14^	7.69^-13^
	ENSG00000286647	*–*	5	6.15	9.57^-13^	3.32^-11^
	ENSG00000261644	*CYLD-AS1*	16	6.06	1.76^-13^	7.47^-12^
**10 most downregulated**	ENSG00000261240	*–*	16	-6.16	4.45^-09^	4.77^-08^
	ENSG00000260573	*–*	16	-5.05	2.53^-07^	1.57^-06^
	ENSG00000249201	*TERLR1*	5	-4.60	4.18^-16^	3.44^-14^
	ENSG00000246985	*SOCS2-AS1*	12	-4.07	3.37^-12^	9.86^-11^
	ENSG00000227487	*NCAM1-AS1*	11	-3.34	6.98^-08^	5.14^-07^
	ENSG00000225649	*–*	2	-3.29	2.89^-15^	1.99^-13^
	ENSG00000266036	*SLC9A3R1-AS1*	17	-3.21	5.51^-10^	7.97^-09^
	ENSG00000224186	*PITX1-AS1*	5	-3.16	1.44^-07^	9.71^-07^
	ENSG00000272549	*LINC02538*	6	-2.97	6.8^-07^	3.7^-06^
	ENSG00000130600	*H19*	11	-2.92	1.58^-14^	8.87^-13^

The fact that we failed to observe any functional and signaling effects of IL-1β and TNFα in EndoC-βH5 cells, prompted us to retrieve information about the basal expression of the receptors for the cytokines used from the RNA-Seq data. Retrieval of the TPM values for interleukin 1 receptor, type 1 (*IL1R1*) and 2 (*IL1R2*), interleukin 1 receptor accessory protein (*IL1RAP*), and TNF receptor superfamily member 1A (*TNFRSF1A*) and 1B (*TNFRSF1B*) revealed very low expression levels with TPM values <1 (**Supplementary Table S1**). In contrast, the IFNγ receptors *IFNGR1* and *IFNGR2* were expressed at a higher level with TPM values of ~3-10. TPM values of key beta-cell identity genes (*INS*, *PDX1*, *MAFA*, and *NKX6-1*) are included in the **Supplementary Table S1** for comparison. A low expression level of *IL1R1* compared to *IFNGR1* was confirmed by qPCR, which revealed Ct values of 35-38 for *IL1R1* and Ct values of 31-33 for *IFNGR1* (data not shown).

Using the IPA tool, we were able to predict the top relevant disease-related and molecular interactions of the differentially expressed genes after exposure to cytokines. The Core analysis revealed that the top-affected canonical pathway was ‘Senescence Pathway’, and the top-affected molecular and cellular function was ‘Cell Death and Survival’ (**Table 2**). Most significant upstream regulators, diseases, and mechanisms of the differentially expressed genes are represented in a graphical summary network (**Figure 5B**). Activated pathways in this network included ‘Diabetes mellitus’, ‘Senescence Pathway’, ‘Endocrine pancreatic dysfunction’, ‘Interferon Signaling’, and ‘Organismal death’.

**Table 2:** Top canonical pathways and top molecular and cellular functions predicted for the differentially expressed genes from the RNA sequencing data using Ingenuity Pathway Analysis (IPA).

Top canonical pathways	p-value	Overlap
Senescence Pathway	1.28*10^-9^	34.4% (103/299)
Kinetochore Metaphase Signaling Pathway	1.27*10^-8^	43.2% (48/111)
Molecular Mechanisms of Cancer	9.04*10^-8^	30.0% (135/450)
Axonal Guidance Signaling	1.09*10^-7^	29.3% (149/509)
Protein Kinase A Signaling	2.12*10^-7^	30.2% (124/411)
Molecular and Cellular Functions	p-value range	Molecules
Cell Death and Survival	1.17*10^-10^ – 3.16*10^-33^	1566
Cellular Assembly and Organization	1.12*10^-12^ – 4.21*10^-29^	827
Cellular Function and Maintenance	8.37*10^-16^ – 4.21*10^-29^	1317
Cellular Movement	8.07*10^-11^ – 1.20*10^-26^	1157
Cellular Development	3.83*10^-11^ – 3.05*10^-26^	1640

The table shows the p-value or p-value range, as well as the overlapping targets identified for each pathway/function, as predicted by the Core analysis in the IPA software.

Finally, we extracted gene expression data of pinpointed candidate genes from 152 type 1 diabetes-associated loci and identified 42 candidate genes that were differentially expressed upon cytokine exposure. Twenty of these were significantly upregulated and 22 significantly downregulated (**Figure 5C**). The data obtained demonstrate that cytokine treatment causes profound changes to the transcriptome of EndoC-βH5 cells.

## 4 Discussion

In the present study, we characterized the sensitivity profile and cellular responses of human EndoC-βH5 cells to diabetogenic cytokines which is a widely used *in vitro* model of beta-cell destruction in diabetes research (12–14). While IL-1β, IFNγ and TNFα have all been detected in islet infiltrates from human donors with type 1 diabetes (10, 11), most of our understanding of their effects and functional pathobiology in diabetes comes from rodent model systems (1, 38). The exact implication and role of these cytokines in human disease thus remain to be fully elucidated. Of note, cytokines are also involved in causing beta-cell dysfunction in type 2 diabetes (15). Hence, although our study focused on cytokine-induced beta-cell impairment relevant for type 1 diabetes, the results should also be of relevance for type 2 diabetes. The observed time- and dose-dependent increase in caspase-3/7 activity and cytotoxicity in EndoC-βH5 cells in response to cytokines is in agreement with studies on EndoC-βH1 cells (3) and human pancreatic islets (25). Interestingly, our study identified IFNγ as the sole cytotoxic cytokine. As shown by immunoblotting, the level of the downstream mediator of IFNγ, P-STAT1, was significantly elevated by IFNγ, whereas P-JNK and IκBα downstream of IL-1β and TNFα receptor signaling, were unaffected by cytokine treatment – at least within the time window examined (30 min). Thus, IFNγ seems to be the main driver of apoptosis signaling in EndoC-βH5 cells. This contrasts with what has been reported for e.g., EndoC-βH1 cells, which respond to IL-1β alone and to a lesser extent TNFα alone with increased apoptosis (3).

The importance of interferons, both type I and II, as diabetogenic cytokines has been stressed by many studies previously, emphasizing their essential roles in insulitis, beta-cell targeting and presence during disease development (34, 39–41). Their role in diabetes was recently highlighted in a study by Apaolaza and colleagues, who investigated the IFN type I (IFNα/β) signature in islets from human donors that were autoantibody positive or recently diagnosed with type 1 diabetes (42). The study reported elevated expression of several markers belonging to the IFN response pathway in islets with insulitis and from donors with autoantibodies or type 1 diabetes, as compared to healthy control islets. Another study reported increased expression of several genes typically induced by type I and II IFNs including *STAT1*, *CXCL10* and *HLA-I* in islet tissue of living individuals with recently diagnosed type 1 diabetes (31, 43). In pancreatic tissue from recent-onset type 1 diabetes patients, a positive correlation between islet cell staining of HLA-A/B/C and STAT1 was observed (31). As STAT1 is activated by both type I and II interferons it can only be speculated whether it is IFNα/β or IFNγ or both that were responsible for these effects. Further studies aimed at defining the pathophysiological role of IFNγ in type 1 diabetes are warranted and may identify rational points of intervention to halt beta-cell killing.

By TUNEL assay, we observed a basal cell death rate for EndoC-βH5 cells of around 20%, which may foremost represent the non-viable cell fraction observed following thawing of the cells. This rate is higher as compared to what is typically observed for other beta-cell models, which have basal death rates of 5-10% (17, 44, 45). This may explain the rather modest ~2-fold cytokine effect on cell death observed in EndoC-βH5 cells. The higher basal death might reflect a more fragile phenotype of the EndoC-βH5 cells compared to other beta-cell lines, which are immortalized and generally exhibit a less differentiated phenotype, including the EndoC-βH1 cell line (44). For human islets, basal cell death varies significantly among studies, between 10-20% (45–47). This may reflect that EndoC-βH5 cells resemble primary human beta cells more closely than other beta-cell lines.

A key characteristic of cytokine-exposed beta cells is the upregulation of immune-modulatory proteins and antigen presentation (18, 32). The MHC class I molecules (encoded by *HLA*) play a pivotal role in the recognition of beta cells by autoreactive T cells (18, 31). We showed that MHC-I was upregulated by cytokines at both the protein and mRNA level. Furthermore, several other beta-cell-produced factors have immune-modulatory effects, including chemoattraction and inflammation (14, 32). Accordingly, we show that the EndoC-βH5 cells secreted several chemokines in response to cytokines. This contributes to their diabetogenic phenotype following cytokine exposure and highlight the use of the EndoC-βH5 cells as a valid model to study the beta cell-immune cell crosstalk in terms of chemokine production. From single cytokine exposure experiments, we found that chemokine secretion was exclusively driven by IFNγ, which verifies that the diabetogenic effects arise from the IFNγ exposure.

One of the main reasons for choosing EndoC-βH5 cells over other beta-cell models is their reported higher insulin content and superior insulin secretory capacity (1, 3, 4, 48). We found that upon stimulation with high glucose, the EndoC-βH5 cells responded with a more than 6-fold induction in insulin release compared to low glucose, which is in agreement with recently published studies (8, 9). The insulin secretory capacity was diminished by cytokine exposure, in accordance with findings in human and rodent beta-cell lines and isolated pancreatic islets (3, 49–52). The cytokine-mediated blunted insulin secretion was observed after 48 hours of cytokine exposure i.e., at a time point before increased cell death was observed, underlining that cytokine-induced functional impairment precedes the induction of cell death.

Our data on mitochondrial function showed reduced respiration following treatment with cytokines. Surprisingly, on several mitochondrial parameters including basal respiration, glucose oxidation, proton leak, and ATP production, combinations of cytokines had the most pronounced inhibitory effects. This observation suggests some degree of synergism between the cytokines with regard to mitochondrial impairment. The underlying mechanisms of cytokine-mediated impaired mitochondrial function are currently unclear but would be relevant to address in future studies. We observed that the glucose response was slightly but significantly increased in EndoC-βH5 cells after exposure to cytokine combinations. This may reflect an increased level of oxidative stress, which is also known to be involved in beta-cell dysfunction (53). Consistent with this notion, retrieval of RNA-Seq data for manganese superoxide dismutase (*SOD2*), an essential mitochondrial antioxidant enzyme, showed a modest but significant upregulation of this gene in response to cytokine treatment (data not shown), supportive of an increased oxidative stress level. Induction of *SOD2* is known to be highly NFκB-dependent (54) and, hence, foremost induced by IL-1β in insulin-secreting cells, however, IFNγ has been found to potentiate IL-1β-induced *SOD2* promotor activity (55). Further, in some cell types, IFNγ alone is sufficient to induce *SOD2* expression (56) underlining that in EndoC-βH5 cells, IFNγ might be sufficient to stimulate weak *SOD2* expression.

Around 50% of the genetic risk of type 1 diabetes resides within the *HLA* region (57). The remaining risk loci harbor both protein-coding and non-coding genomic regions, emphasizing the implication of both in type 1 diabetes risk (24, 57). By RNA-seq analysis, we found that 42 pinpointed candidate risk genes from type 1 diabetes-associated loci were differentially expressed after cytokine exposure. These included both mRNAs and lncRNAs. Overall, we detected more than 16,000 genes expressed in EndoC-βH5 cells of which more than a third (6,000 genes: 1,934 up; 4,066 down) were modified by cytokines. This differential expression is seemingly larger than what was previously reported in similar studies of human islets and Endo-βH1 cells (58, 59), and may represent cell model-specific variations but could also result from experimental differences. By pathway analysis, we were able to predict relevant molecular interactions, including functions related to cell death and survival, and diabetes. Furthermore, we validated several mRNAs and lncRNAs that are known to be implicated in beta-cell apoptosis and dysfunction (18, 19), highlighting that despite EndoC-βH5 cells in some aspects appear different from e.g. EndoC-βH1 cells, they also share important characteristics with more established beta-cell models.

We found very low expression levels of the receptors for IL-1β and TNFα questioning whether EndoC-βH5 cells are sensitive to IL-1β and TNFα at all. In contrast, the receptor for IFNγ, *IFNGR1/2*, was expressed at a higher level. The low expression of the IL-1 and TNF receptors provides a plausible explanation as to why IL-1β and TNFα failed to show individual or synergistic effects when combined with IFNγ. Although very low expression of particularly *IL1R1* may be a characteristic feature of EndoC-βH5 cells, this receptor also seems lowly expressed relative to the IFNγ receptor in both EndoC-βH1 cells (59) and purified human beta cells (60, 61) as revealed by extraction of available RNA-Seq data from these cells. The low expression levels of the IL-1 and TNF receptors in EndoC-βH5 cells may be a limitation of this cell model regarding the use of these cells for studies using other cytokines than IFNγ.

Thus far, only few studies have been published on EndoC-βH5 cells (8, 9), but the cells hold promise as a superior model of native human beta cells over other human beta-cell models. The validity of the human hybrid beta-cell line 1.1B4 (62) must be questioned, as a study reported rodent cell contamination in the cell stocks and established deprivation of insulin and glucose responsiveness after isolation of the human cell population (48). The EndoC-βH1 cell line is the most widely used human beta-cell line and has been used by many research laboratories for the past 10 years (2–4). However, EndoC-βH1 cells suffer from functional limitations compared to native beta cells (3–7). Moreover, during the original expansion of the EndoC-βH1 cells in mice, the cells were stably infected with a xenotropic murine virus (5). It is still uncertain to which extent the infection with this virus affects the cells’ phenotype and cautions should be taken when extrapolating results from this cell line.

Although of relevance, we chose not to perform a direct head-to-head comparison of the EndoC-βH5 cells with other cell models. The present work is therefore limited with regards to determining the exact discrepancies between EndoC-βH5 cells and e.g., human islets and EndoC-βH1 cells. However, our study offers detailed insight into the EndoC-βH5 cells’ cytokine sensitivity profile and cellular responses including functional and transcriptomic effects. Besides being advantageous by having non-cancerous properties, i.e., a non-proliferative phenotype, and absence of the xenotropic murine virus, EndoC-βH5 cells possess key beta-cell properties including a high insulin secretory capacity. We demonstrate the EndoC-βH5 cells’ selective responsiveness to IFNγ over other diabetogenic cytokines, highlighting IFNγ as the most potent cytokine in this beta-cell model. Although this appears to be a potential limitation of the EndoC-βH5 cells and a trait not necessarily representative of native human beta cells, further studies are required to fully establish if other diabetogenic cytokines including IL-1β and TNFα have deleterious effects in EndoC-βH5 cells. Our study demonstrates that IFNγ induces several relevant cellular responses including upregulation of MHC-I, STAT1 activation, and chemokine secretion. To our knowledge, this is the first study describing the EndoC-βH5 cells’ sensitivity profile to cytokines. It will be important to further investigate IFNγ-induced responses in beta cells to decipher the underlying mechanisms of this key diabetogenic factor to understand it’s role in a pathophysiological context in humans.

## Data availability statement

The data presented in the study are deposited in the Gene Expression Omnibus (GEO) repository, accession number GSE218735.

## Author contributions

CF and JS conceptualized the study. CF, RG, FP, SK, and JS designed the study. CF, RG, CS, JM, KH, TF, and SK contributed to the acquisition of data. CF made the first draft of the manuscript which was commented by JS. All authors contributed to the article and approved the submitted version.
