# Supplementary material for: Characterization of the functional and transcriptomic effects of pro-inflammatory cytokines on human EndoC-βH5 beta cells
Source: Front Endocrinol (Lausanne). 2023 Apr 11;14:1128523. doi: 10.3389/fendo.2023.1128523 (PMC10126300; doi:10.3389/fendo.2023.1128523)
Supplement: Supplementary file 1 [file DataSheet_1.docx]

Supplementary Material

Characterization of the functional and transcriptomic effects of pro-inflammatory cytokines on human EndoC-βH5 beta cells

**Caroline Frørup^1^, Rebekka Gerwig^1^, Cecilie Amalie Søndergaard Svane^1^, Joana Mendes Lopes de Melo^1^, Kristine Henriksen^1^, Tina Fløyel^1^, Flemming Pociot^1,2^, Simranjeet Kaur^1^, Joachim Størling^1,3*^**

^1^Translational Type 1 Diabetes Research, Clinical Research, Steno Diabetes Center Copenhagen, Herlev, Denmark

^2^Faculty of Health and Medical Sciences, University of Copenhagen, Copenhagen, Denmark

^3^Department of Biomedical Sciences, University of Copenhagen, Copenhagen, Denmark

*** Correspondence:** Joachim Størling: joachim.stoerling@regionh.dk

# Supplementary Data

## Supplementary Tables

**Supplementary Table S1** TPM values of cytokine receptors and beta-cell identity genes retrieved from RNA sequencing of untreated control cells or cells exposed to cytokines (50 U/mL IL-1β, 1000 U/mL IFNγ and 1000 U/mL TNFα) for 48 h (CTRL_1-4, 4 replicates of untreated control cells; CYT_1-4, 4 replicates of cytokine-exposed cells).

| **Gene ID** | **Gene name** | **Chr.** | **CTRL_1** | **CTRL_2** | **CTRL_3** | **CTRL_4** | **CYT_1** | **CYT_2** | **CYT_3** | **CYT_4** |
| --- | --- | --- | --- | --- | --- | --- | --- | --- | --- | --- |
| ENSG00000115594 | *IL1R1* | 2 | 0.02 | 0.02 | 0.03 | 0.04 | 0.03 | 0.04 | 0.05 | 0.06 |
| ENSG00000115590 | *IL1R2* | 2 | 0.02 | 0.01 | 0.03 | 0.05 | 0.00 | 0.01 | 0.02 | 0.11 |
| ENSG00000196083 | *IL1RAP* | 3 | 0.14 | 0.14 | 0.20 | 0.20 | 0.23 | 0.21 | 0.33 | 0.30 |
| ENSG00000067182 | *TNFRSF1A* | 12 | 0.40 | 0.50 | 0.61 | 0.54 | 0.77 | 0.78 | 0.90 | 0.90 |
| ENSG00000028137 | *TNFRSF1B* | 1 | 0.08 | 0.00 | 0.00 | 0.05 | 0.06 | 0.02 | 0.13 | 0.18 |
| ENSG00000027697 | *IFNGR1* | 6 | 3.75 | 4.16 | 6.72 | 5.36 | 6.83 | 6.18 | 9.05 | 9.97 |
| ENSG00000159128 | *IFNGR2* | 21 | 2.57 | 2.75 | 5.55 | 4.35 | 3.23 | 3.24 | 6.65 | 5.42 |
| ENSG00000254647 | *INS* | 11 | 10608.0 | 13137.7 | 23653.1 | 12251.5 | 11158.2 | 10638.7 | 23514.2 | 15199.3 |
| ENSG00000139515 | *PDX1* | 13 | 178.89 | 182.51 | 288.64 | 218.21 | 132.93 | 128.51 | 187.16 | 153.15 |
| ENSG00000182759 | *MAFA* | 8 | 3.64 | 4.12 | 10.02 | 6.82 | 0.93 | 1.07 | 2.42 | 1.60 |
| ENSG00000163623 | *NKX6-1* | 4 | 66.78 | 60.70 | 108.50 | 68.22 | 103.97 | 99.30 | 175.19 | 100.05 |

## Supplementary Figures

**
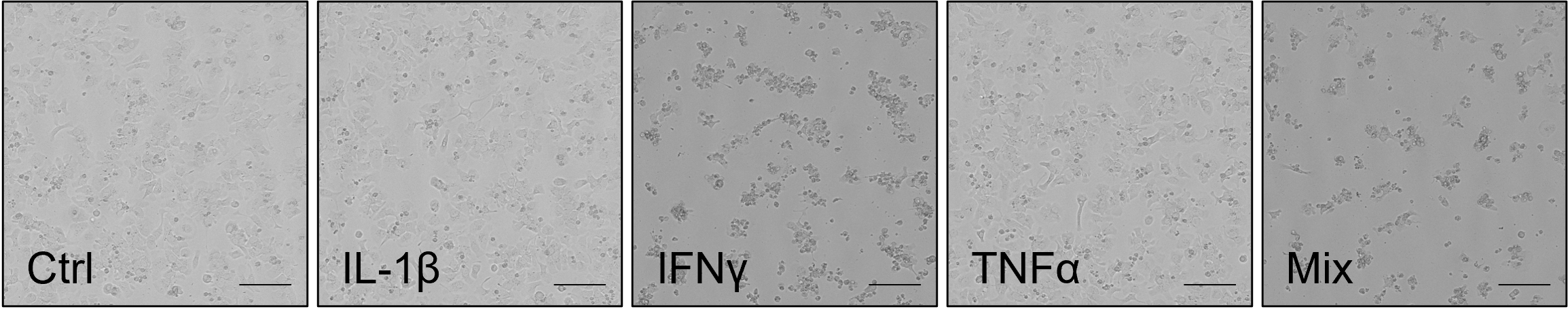
**

**Supplementary Figure S1** Representative phase contrast images of EndoC-BH5 cells left untreated (Ctrl) or after 7 days of cytokine treatment (50 U/mL IL-1β, 1000 U/mL IFNγ and 1000 U/mL TNFα) in combination (Mix) or individual cytokines, respectively. Scale bars indicate 200 μm.


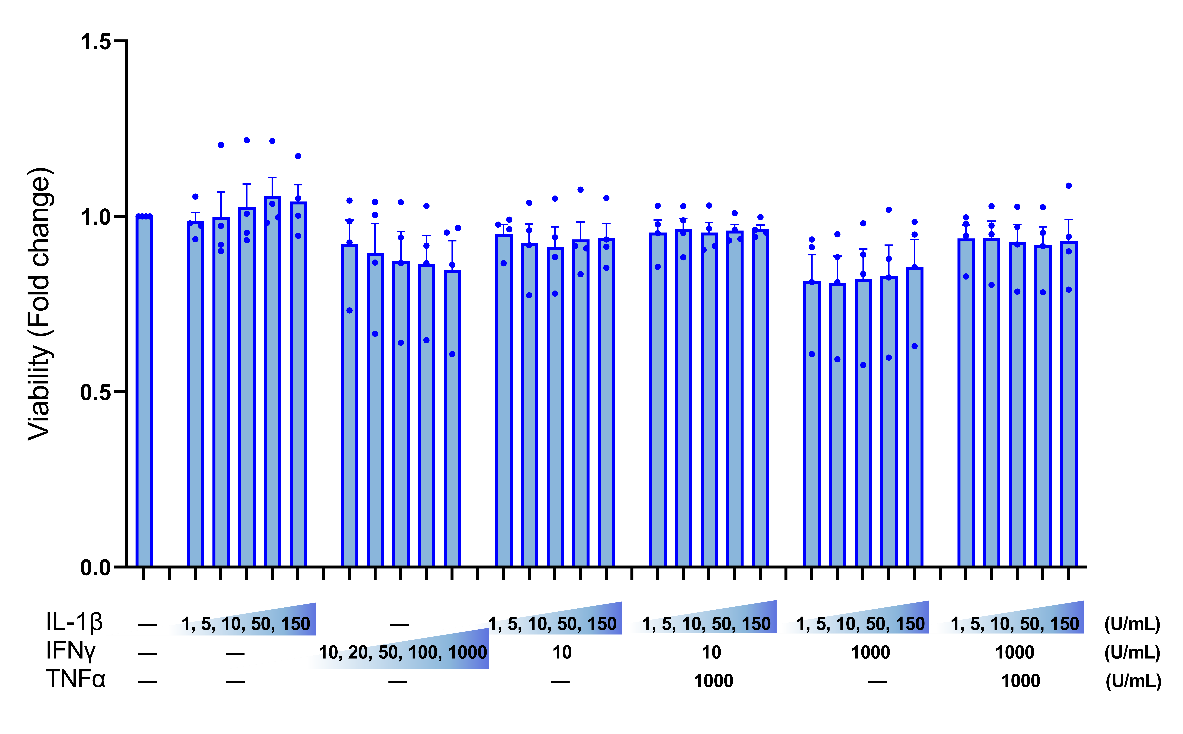


**Supplementary Figure S2** Viability after exposure to IL-1β, IFNγ and TNFα at the indicated concentrations for 96 h. Data are means ± SEM (n=4).

A B C D


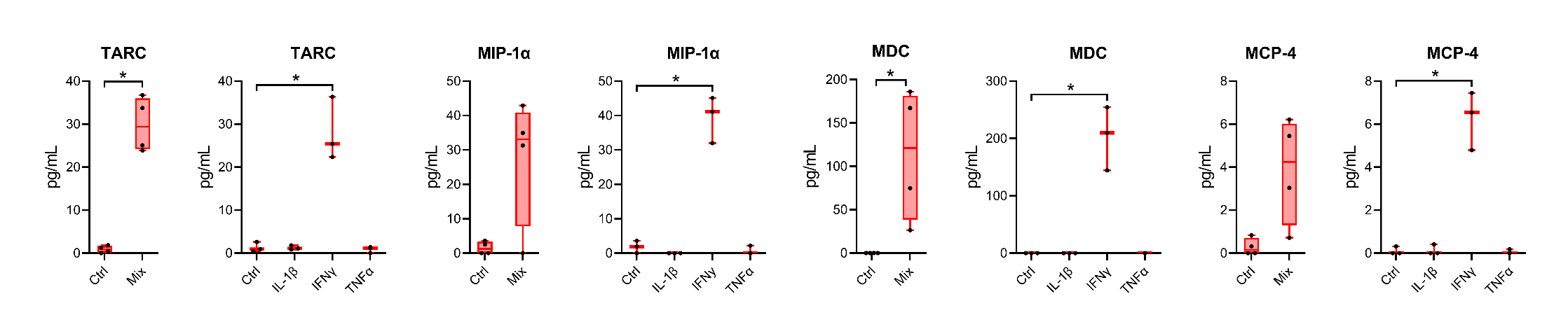


E F G H


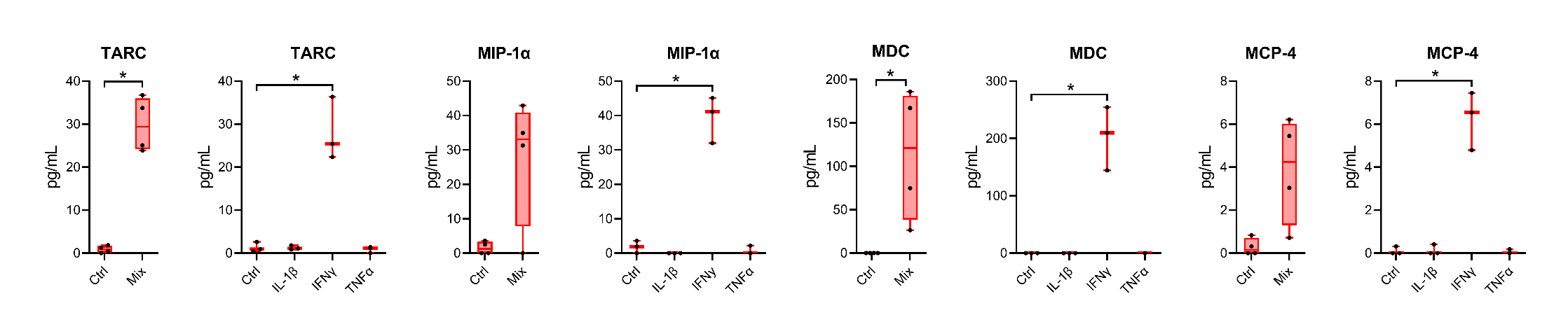


**Supplementary Figure S3** Accumulated chemokines in the culture medium from EndoC-βH5 cells after 48 h of cytokine treatment (50 U/mL IL-1β, 1000 U/mL IFNγ and 1000 U/mL TNFα) in combination (Mix) or individual cytokines, respectively: (**A**-**B**) TARC/CCL17 (**C**-**D**) MIP-1α/CCL4, (**E**-**F**) MDC/CCL22, (**G**-**H**) MCP-4/CCL13. Data are mean pg/mL with median and 5/95 percentiles (n=3-4).

A B


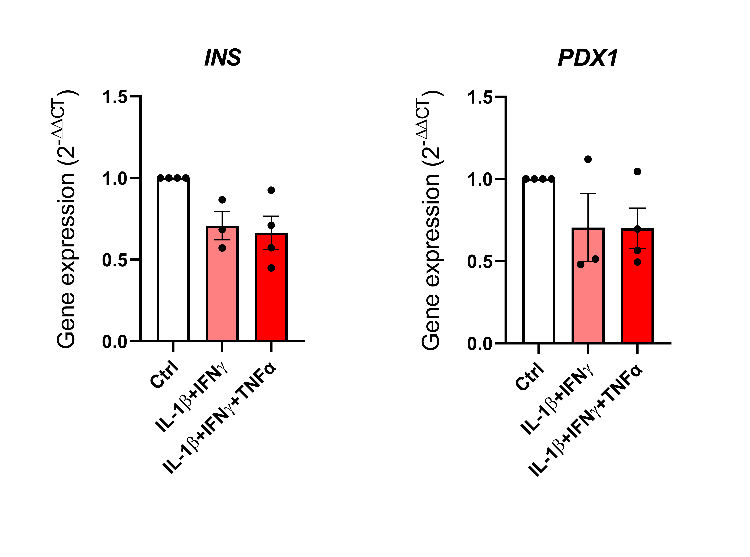


**Supplementary Figure S4** Expression of (**A**) *INS* and (**B**) *PDX1* after exposure to cytokines (50 U/mL IL-1β, 1000 U/mL IFNγ and 1000 U/mL TNFα) for 48 h. *GAPDH* was used as housekeeping gene. Data are means ± SEM (n=3-4).

A B

**
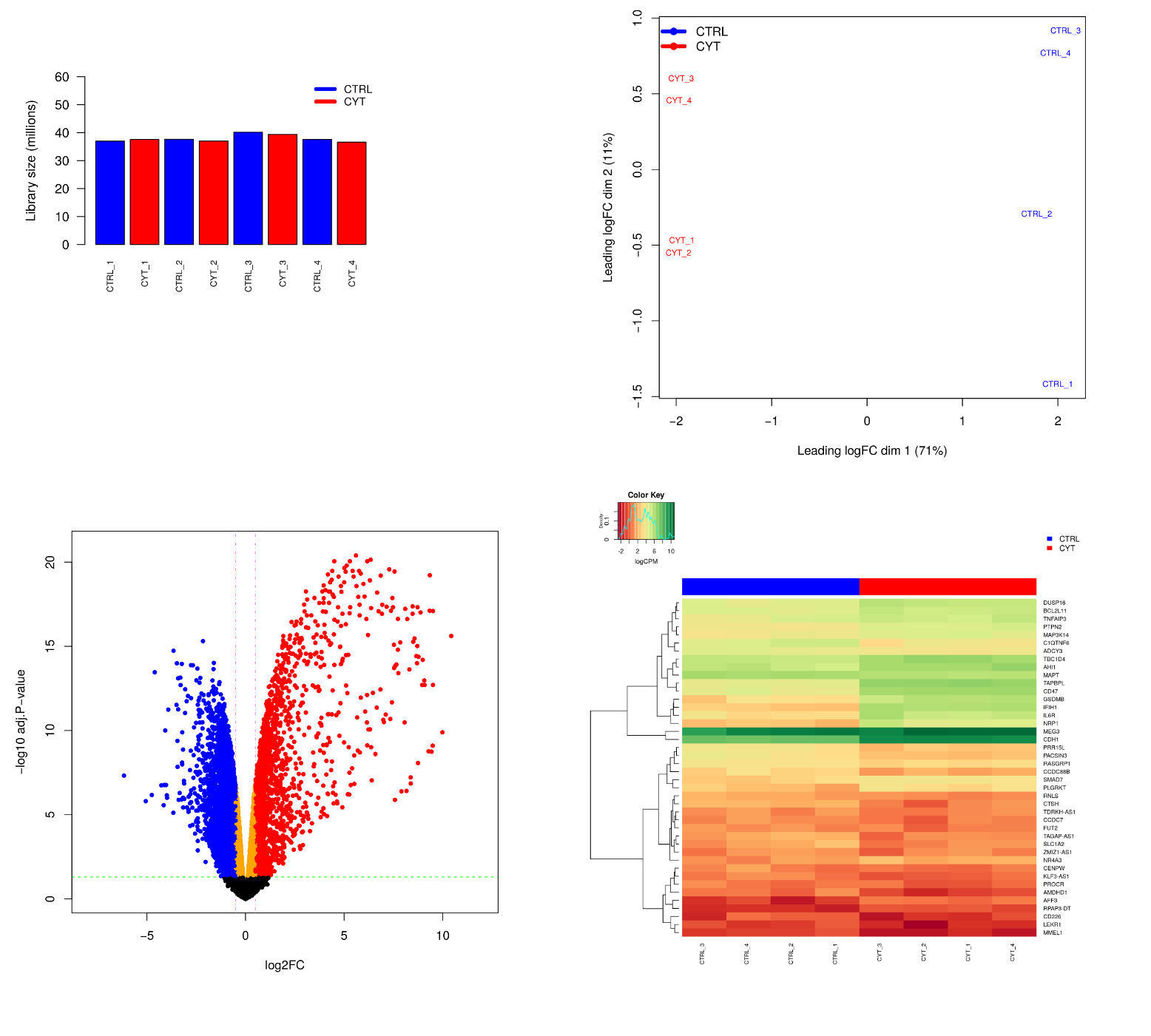
**
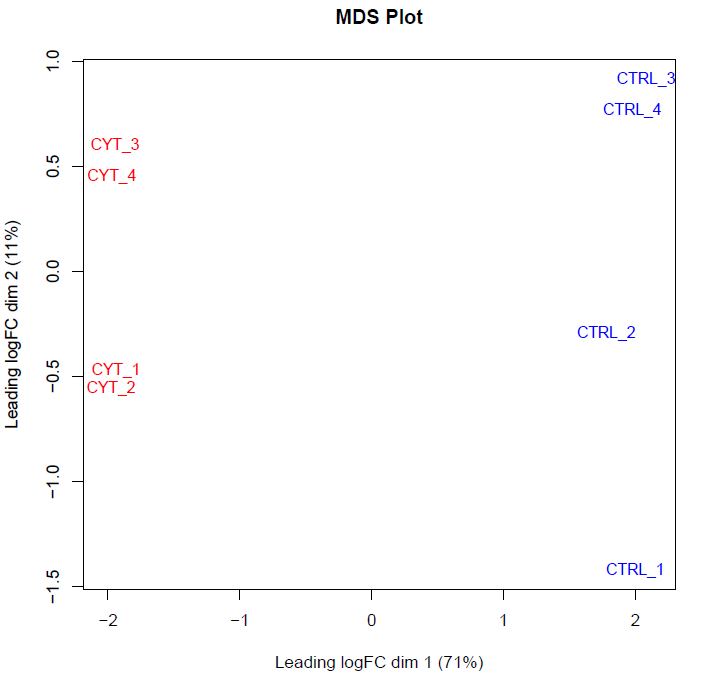


**Supplementary Figure S5** RNA sequencing of untreated control cells or cells exposed to cytokines (50 U/mL IL-1β, 1000 U/mL IFNγ and 1000 U/mL TNFα) for 48 h. (**A**) Library size and (**B**) MDS plot of each of the RNA samples sequenced (CTRL_1-4, 4 replicates of untreated control cells; CYT_1-4, 4 replicates of cytokine-exposed cells).

A B C D

**
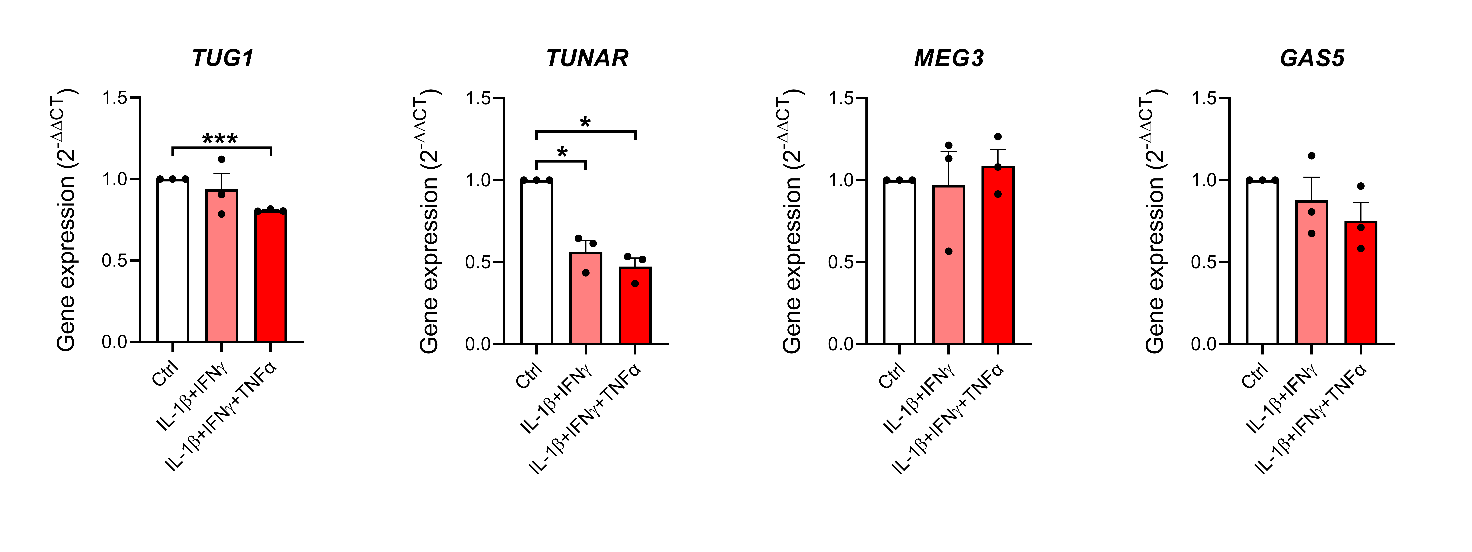
 Supplementary Figure S6** Expression of four lncRNAs (**A**) *TUG1*, (**B**) *TUNAR*, (**C**) *MEG3* and (**D**) *GAS5* after exposure to cytokines (50 U/mL IL-1β, 1000 U/mL IFNγ and 1000 U/mL TNFα) for 48 h. *GAPDH* was used as housekeeping gene. Data are means ± SEM (n=3), *p<0.05, ***p<0.001.
